# *Streptomyces bikiniensis* Bacteremia

**DOI:** 10.3201/eid0902.020275

**Published:** 2003-02

**Authors:** William J. Moss, Jason A. Sager, James D. Dick, Andrea Ruff

**Affiliations:** * Johns Hopkins University, Baltimore, Maryland

**Keywords:** *Streptomyces*, bacteremia, letter

**To the Editor:** Carey et al. recently reported in this journal a case of catheter-related bacteremia attributed to *Streptomyces* in a patient receiving holistic infusions (*1*). We describe the isolation of *Streptomyces bikiniensis* from multiple blood cultures in a single patient over the course of 1 week, further illustrating that *Streptomyces* is pathogenic and a cause of bacteremia even in the absence of overt clinical symptoms and risk factors.

A 14-year-old girl with osteosarcoma of the right proximal tibia came to our hospital 13 months after diagnosis for her final course of chemotherapy. At the time of diagnosis, a double-lumen central venous catheter was inserted. Her course was complicated by poor response to chemotherapy, and a limb salvage procedure was performed 3 months after diagnosis. The proximal tibia was replaced with a cadaveric bone graft. Several hours after the patient received methotrexate, a fever of 39.2°C developed. No sign of infection was observed on physical examination. Her leukocyte count was 6,300 cells/mm^3^ with an absolute neutrophil count of 4,914 cells/mm^3^. She received a single dose of acetaminophen and was without fever for the remainder of her hospitalization. A blood culture obtained from the central venous catheter at the time of fever grew *Streptomyces*. Repeat blood cultures obtained from both ports of the central venous catheter on day 3 and a peripheral blood culture obtained on day 4 also grew *Streptomyces*. Treatment with vancomycin and cotrimoxazole was started on day 4 in the hospital. The *Streptomyces* isolate was susceptible to vancomycin, amikacin, cotrimoxazole, erythromycin, cephazolin, and tetracycline and was resistant to ampicillin, penicillin, oxacillin, and clindamycin. A blood culture drawn from the central venous catheter on day 3 of antibiotic therapy (the 6th day in the hospital) grew *Streptomyces* after 9 days of incubation. All subsequent blood cultures were without growth. The central venous catheter was removed, and the patient received vancomycin intravenously for 6 weeks, without recurrence of *Streptomyces* bacteremia.

The bone graft was considered a potential source of infection. As most cases of disease from *Streptomyces* occur in the tropics, we requested information on whether the donor traveled or resided outside the United States. However, the donor had no history of travel outside the United States. All cultures taken from the donor and the graft were without growth (although this did not exclude the graft as the source of infection), and no reports of disease transmission were received from any other recipients of organs from this donor. In addition, the patient had no history of receiving infusions of holistic or alternative medicines.

The organism was initially detected in the aerobic Bact/Alert blood culture system (bioMérieux, Inc., Durham, NC) after 72 h incubation at 35°C. Presumptive identification of the pleomorphic gram-positive bacillus as *Streptomyces* sp. was based on phenotypic characterization by using standard conventional tests and cellular fatty acid analysis. Species identification was determined by DNA sequencing of the 16S rRNA gene. DNA sequencing reactions were performed with the Tag Dye Deoxy Terminator Cycle Sequencing Kit (Applied Biosystems, Inc., Foster City, CA), and data were generated with an ABI 377 automated instrument. The sequence data were assembled, edited, and compared with published sequences for the 16S rRNA gene of *S. bikiniensis* (*2*).

The genus *Streptomyces* belongs to the order *Actinomycetales*, which includes *Mycobacterium*, *Nocardia*, and *Actinomyces*. *Streptomyces* are gram-positive, extensively branched, filamentous bacteria that form aerial hyphae with chains of spores. Their natural habitat is soil, and each species has a defined geographic distribution. None are common in the United States. With the exception of specimens from actinomycotic mycetoma, the isolation of *Streptomyces* from clinical specimens frequently is considered laboratory contamination (*3*). Rare cases of clinical disease attributed to *Streptomyces* have been published, including bloodstream infection (*1*,*4*) and focal invasive infections (*5*–*9*). *Streptomyces* was not the only potential pathogen isolated from some of the clinical specimens in these studies.

Scant data are available on effective treatment of *Streptomyces* infection. Mycetoma caused by *Streptomyces* is often treated with penicillin, sulfonamides, or tetracycline; however, the cure rate is low. The recommended duration of therapy is lengthy (up to 10 months). Isolates of *S. griseus* referred to the Centers for Disease Control and Prevention were frequently resistant to ampicillin (80%), sulfamethoxazole (43%), cotrimoxazole (29%), and ciprofloxacin (57%) (*10*). Resistance to doxycycline (19%) and minocycline (10%) was lower. Vancomycin susceptibility was not tested. Resistance patterns must be interpreted cautiously because *Streptomyces* can synthesize antibiotics, potentially confounding results of in-vitro susceptibility testing.

The patient described in this report had no signs or symptoms of infection. The transient fever that prompted the first blood culture was probably due to the methotrexate infusion and not infection with *S. bikiniensis*. That the fever was of short duration despite persistently positive blood cultures supports this conclusion. The potential for causing minimal symptoms may contribute to assignment of *Streptomyces* as a contaminant. Clinical correlation is difficult if the infection is silent. *Streptomyces* isolated from blood cultures should not be dismissed as contaminants without careful consideration of the clinical situation; the isolation of *Streptomyces* from repeat blood cultures strongly suggests a pathogenic role.
